# Endothelial cell dynamics in sepsis-induced acute lung injury and acute respiratory distress syndrome: pathogenesis and therapeutic implications

**DOI:** 10.1186/s12964-024-01620-y

**Published:** 2024-04-25

**Authors:** Xinyu Qiao, Junhao Yin, Zhihuan Zheng, Liangge Li, Xiujing Feng

**Affiliations:** 1 Shandong Provincial Key Laboratory for Rheumatic Disease and Translational Medicine, The First Affiliated Hospital of Shandong First Medical University & Shandong Provincial Qianfoshan Hospital, Jinan, China; 2 School of Clinical and Basic Medical Sciences, Shandong First Medical University& Shandong Academy of Medical Sciences, Jinan, 250117 Shandong China; 3 Key Laboratory of Endocrine Glucose & Lipids Metabolism and Brain Aging, Ministry of Education; Department of Endocrinology, Shandong Provincial Hospital Affiliated to Shandong First Medical University, Jinan, 250021 Shandong China

**Keywords:** Sepsis, Sepsis-induced ALI/ARDS, Endothelial cells, Stromal cells, Interactions

## Abstract

Sepsis, a prevalent critical condition in clinics, continues to be the leading cause of death from infections and a global healthcare issue. Among the organs susceptible to the harmful effects of sepsis, the lungs are notably the most frequently affected. Consequently, patients with sepsis are predisposed to developing acute lung injury (ALI), and in severe cases, acute respiratory distress syndrome (ARDS). Nevertheless, the precise mechanisms associated with the onset of ALI/ARDS remain elusive. In recent years, there has been a growing emphasis on the role of endothelial cells (ECs), a cell type integral to lung barrier function, and their interactions with various stromal cells in sepsis-induced ALI/ARDS. In this comprehensive review, we summarize the involvement of endothelial cells and their intricate interplay with immune cells and stromal cells, including pulmonary epithelial cells and fibroblasts, in the pathogenesis of sepsis-induced ALI/ARDS, with particular emphasis placed on discussing the several pivotal pathways implicated in this process. Furthermore, we discuss the potential therapeutic interventions for modulating the functions of endothelial cells, their interactions with immune cells and stromal cells, and relevant pathways associated with ALI/ARDS to present a potential therapeutic strategy for managing sepsis and sepsis-induced ALI/ARDS.

## Introduction

Sepsis represents a form of systemic inflammatory response syndrome triggered by severe infections characterized by systemic dissemination disease and capable of causing multi-organ impairment. Recent epidemiological data from a survey conducted between 2015 and 2016 revealed a notably high 90-day mortality rate of 35.5% for sepsis [1]. Additionally, a multicenter study conducted in Brazil in 2017 reported that one-third of intensive care unit (ICU) beds were occupied by septic patients, with an alarming mortality rate of 55.7% [2]. In current clinical practice, the prompt initiation of early and efficacious antimicrobial treatment, along with the timely administration of vasopressors, are vital components in the management of this condition [3]. Despite the many treatment strategies available in clinical settings aimed at prolonging life and reducing short-term mortality, thus far, there is a lack of highly efficient treatments that can mitigate the adverse events associated with sepsis [4]. Among these, the lung, a vital organ responsible for gas exchange and a significant immune organ defending the host against inhaled pathogens, allergens and xenobiotics (such as in allergic asthma and pneumonia), is the most susceptible organ affected during sepsis [5]. Sepsis-induced acute lung injury (ALI) and its most severe form, acute respiratory distress syndrome (ARDS), are devastating clinical conditions marked by refractory hypoxemia, respiratory distress, and non-cardiogenic pulmonary edema [6, 7]. Clinically, ALI and ARDS represent distinct stages in the same disease process, with ALI representing the early and milder phase, while ARDS signifies the late and often severe stage. Presently, the Berlin definition, established by an expert panel in 2012, serves as the unified standard for ARDS diagnosis. This definition encompasses various criteria, including the timing of onset, chest imaging findings, origin of edema, oxygenation parameters, and other clinical indicators [8]. The key differentiator in the diagnostic process between ALI and ARDS lies in the oxygen partial pressure to inspiratory fraction (PaO_2_/FiO_2_) ratio, whereby ALI is characterized by a PaO_2_/FiO_2_ ratio ≤ 300mmHg and the PaO_2_/FiO_2_ limiting value of ARDS is lower, ≤ 200mmHg [9]. Despite significant advances in the understanding and management of ALI/ARDS, there remains a substantial lack of drugs capable of effectively treating ALI induced by sepsis due to limited research on its underlying mechanisms.

The endothelium, comprising endothelial cells (ECs), forms a monolayer barrier along the inner surface of the vasculature, playing diverse roles in lung pathology and serving as a semi-permeable interface between circulating blood and underlying tissues. In recent years, researchers have increasingly focused on the activation of ECs and their interactions with immune cells and stromal cells in the context of sepsis-induced ALI/ARDS, as they are often related to endothelial barrier disruption [10]. Damage to this barrier, driven by an excessive inflammatory response, results in increased pulmonary vascular permeability. This facilitates the entry of circulating fluids, macromolecules and leukocytes into alveoli, leading to alveolar flooding and neutrophil infiltration, thereby significantly contributing to the elevated mortality associated with ALI/ARDS [11]. In this review, we discuss the significance of ECs and ECs-immune cell and ECs-stromal cell interactions in the pathogenesis of sepsis-induced ALI or ARDS. Additionally, we discuss strategies for targeting ECs and their interactions with immune cells and stromal cells, either directly or indirectly, as potential therapeutic approaches to mitigate the adverse effects of this intercellular communication in the context of this disease.

## Endothelial cell activation and dysfunction in sepsis-induced ALI/ARDS

ECs, situated along the inner lining of blood vessels, play a pivotal role in orchestrating numerous physiological functions, such as regulating blood fluidity, vascular tone, cellular and nutrient transport, and promoting neovascularization under normal conditions [12]. They are not only sensitive to self-produced substances and extracellular matrix components [13] but also activate transcellular and intracellular signaling pathways by secreting molecules in response to various stimuli, thereby contributing to the regulation of hemostasis, vasomotor control and immunological functions [14–16]. In addition, pulmonary ECs, which are responsible for regulating alveolar-capillary interactions, are interconnected through intercellular junctions, such as tight junctions (TJs), gap junctions and adherens junctions (AJs) [17]. TJs between ECs are formed by the outermost plasma membrane and consist of occludins, claudins and junctional adhesion molecules connected to cytoplasmic proteins. These proteins are in turn linked to the ECs’ actin cytoskeleton through the zonula occludens family, and as a result, TJs control endothelial paracellular permeability by regulating the diffusion of fluids, ions, and small plasma proteins, as well as the infiltration of cells such as leukocytes, neutrophils, and lymphocytes. This mechanism effectively establishes a barrier within pulmonary blood vessels [18]. In addition, AJs are composed of calcium-dependent cadherins, with vascular endothelial cadherin (VE-cadherin) being the primary cadherin involved. VE-cadherin binds to intracellular catenin proteins, which, in turn, interact with other protein partners within the actin cytoskeleton. AJs are also essential regulatory elements governing the paracellular transport of cells and solutes between the bloodstream and the interstitium, which significantly influences endothelial cell permeability, white blood cell migration, and the formation of edema, among other essential functions [19].

Endothelial activation refers to the response of ECs to various stimuli, including hypoxia, cytokines such as TNF-α and IL-1β, chemokines, thrombin and bacterial endotoxin (LPS), as well as interactions with inflammatory cells. Typically, endothelial activation is initiated by the interaction of LPS with pattern recognition receptors on the surface of ECs, which include Toll-like receptors (TLRs), Pathogen-Associated Molecular Patterns (PAMPs) and Damage-Associated Molecular Patterns (DAMPs) [20]. Among these receptors, TLR4 is the primary receptor for LPS expressed in ECs, and its activation by LPS leads to the modulation of inflammatory cytokines. This activation process forms the basis for phenotypic transitions and other functional changes in ECs, involving a shift from a resting state to a pro-inflammatory and coagulant phenotype, which promotes adhesion and increases oxidative stress [21]. Increased expression or release of EC adhesion molecules and other cytokines, along with the upregulation of proinflammatory transcription factors, represent the most typical hallmark of endothelial activation [22], such as the Nuclear factor of the kappa light chain (NF-κB pathway) [23, 24]. Previous studies have shown that depleting Yes-associated protein (YAP) in ECs significantly enhances the inflammatory response in a cecal ligation and puncture (CLP)-induced sepsis model, highlighting the role of tumor necrosis factor receptor-associated factor 6 (TRAF6)-mediated activation of the NF-κB pathway in regulating EC activation [25]. Additionally, recent research has reported that TRIM47, an E3 ubiquitin ligase highly expressed in ECs, activates NF-κB and mitogen-activated protein kinase (MAPK) signaling pathways through K63-associated TRAF2 ubiquitination to promote LPS-induced lung inflammation and the development of ALI/ARDS in ECs [26].

Endothelial dysfunction is characterized by a pro-inflammatory state, impaired vasodilation and increased propensity for thrombosis within the endothelium. In the context of sepsis, severe endothelial dysfunction leads to disturbances in hemostasis, aberrant vascular reactivity, and tissue edema. Specifically, in sepsis-induced ALI/ARDS, ECs can release inflammatory mediators after EC activation and recruit leukocytes, which increase their adhesion to the vascular endothelium and infiltrate into deeper layers. Once leukocytes, particularly neutrophils and monocytes, enter the lung parenchyma, they exacerbate an imbalance between pro-inflammatory and anti-inflammatory responses due to an over-activated immune reaction within the lungs, leading to a cytokine storm and damage to vascular and lung tissues [26, 27]. Moreover, excessive activation of ECs can trigger coagulation in an attempt to isolate infections. The resulting endothelial damage from this excessive activation promotes procoagulation and increased permeability, resulting in capillary thrombosis, disseminated intravascular coagulation (DIC), pulmonary edema and pulmonary hemorrhage [28]. The adhesion of leukocytes to ECs and their subsequent migration across ECs, mediated by these factors, can stimulate ECs to adopt anti-inflammatory and other functions, ultimately leading to EC dysfunction. Therefore, while ECs initially respond to various stimuli by modifying the release of adhesion molecules and other cytokines, if the level of endothelial activation surpasses a certain threshold, ECs progress into a state of dysfunction, further compromising the integrity of the endothelial barrier. Throughout this entire process, ECs adapt their phenotype and function, including coagulation and pro-inflammatory responses, in an effort to regulate the pulmonary microenvironment.

### Glycocalyx damage

**Fig. 1:**
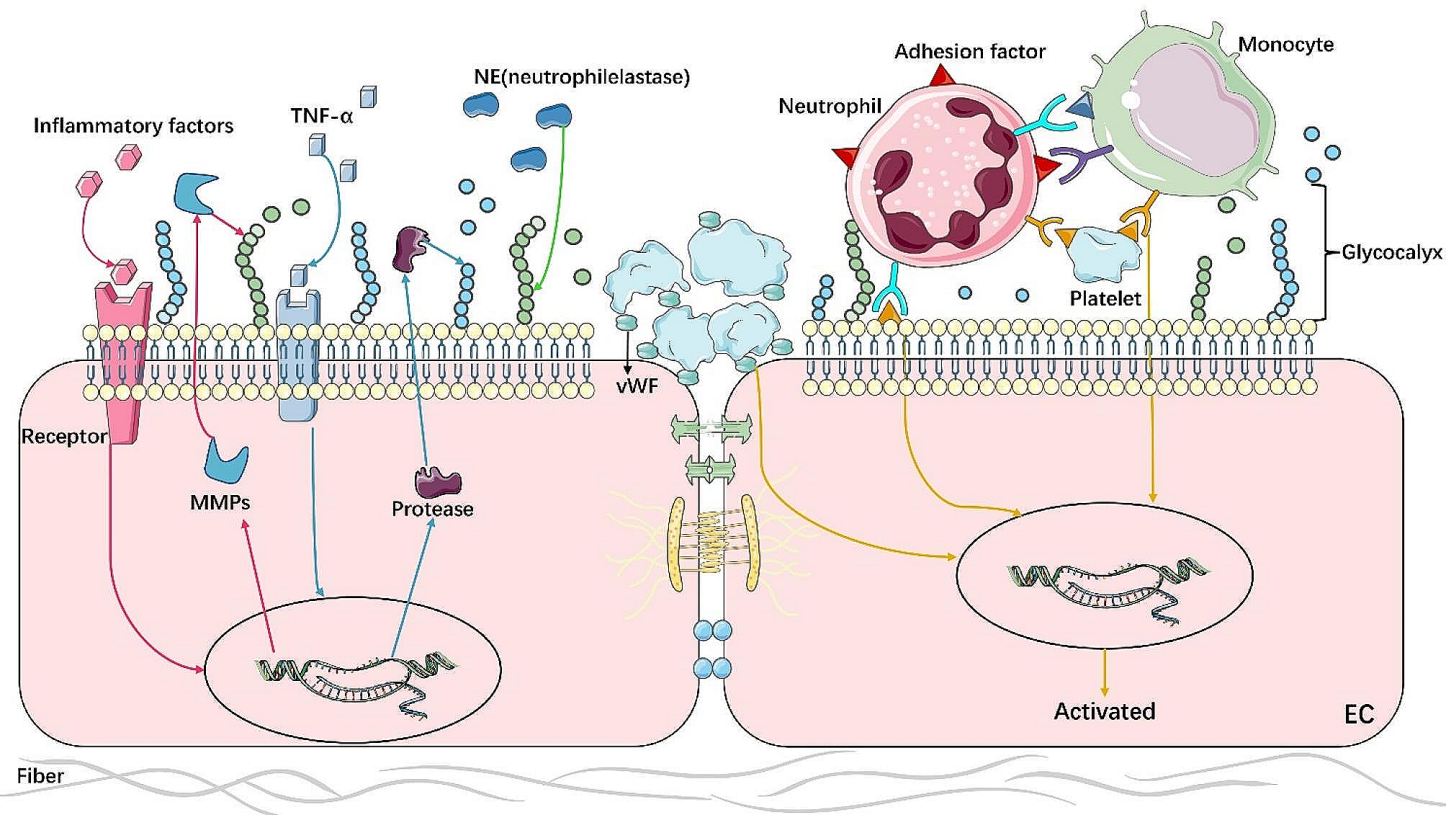
**Mechanism of glycocalyx degradation in LPS or septic conditions. **Inflammatory factors mediate the expression of MMPs to induce glycocalyx degradation. TNF-α acts on ECs to express protease or nucleotide enzyme, inducing glycocalyx degradation. Neutrophil proteinases released by neutrophils act on glycocalyx, directly causing its degradation. Glycocalyx degradation exposes adhesion factors on the surface of ECs that induce adhesion and interaction of platelets and leukocytes on the surface of ECs

ECs are enveloped by a layer of glycocalyx, strategically positioned to interact with blood-borne cells and vasoactive mediators, enabling them to perceive mechanical, chemical, and cellular stimuli [29]. The endothelial glycocalyx comprises three main components: membrane-binding proteoglycans (PGs) (such as syndecan and glypican), glycosaminoglycan (GAG) side chains attached to the core protein of proteoglycans, and plasma proteins (such as albumin and antithrombin) [30]. Additionally, GAG within the glycocalyx can bind to various substances, such as hyaluronic acid (HGAG within the glycocalyx can bind to various substances, such as hyaluronic acid (HA) and thrombomodulin (TM) and thrombomodulin (TM), to stabilize the glycocalyx [31]. In sepsis-induced ALI, the glycocalyx on ECs undergoes degradation or shedding, exposing signal receptors on the endothelial surface. Syndecan-1, a crucial biomarker for glycocalyx integrity, is released into the bloodstream upon glycocalyx degradation [32]. Hence, syndecan-1 serves as a hallmark of ALI/ARDS, and the measurement of its serum levels can predict the progression of ALI/ARDS in patients [33]. Using both electron and fluorescence intravital microscopy, researchers observed that the thickness of the endothelial glycocalyx in septic mice measured only 0.98 nm, in contrast to 70.68 nm in matched control subjects [34]. In line with these findings, Inagawa et al. reported severe disruption, peeling, and coagulation of the endothelial glycocalyx, which normally appears as a “moss-like structure” in LPS-induced mice [32]. Current studies have revealed that endothelial glycocalyx degradation during sepsis occurs via inflammatory mechanisms involving heparinase, metalloproteinases, and hyaluronidase [35] (Fig. 1).

Heparinase, classified as an endoglycosidase, plays a pivotal role in cleaving heparan sulfate (HS) within glycocalyx, contributing to the degradation and remodeling of the extracellular matrix [36]. It is activated in sepsis-induced ALI/ARDS, leading to the degradation of HS moieties [37]. Heparinase-1, the sole identified mammalian enzyme capable of breaking down HS polysaccharides into shorter-chain oligosaccharides, represents the only known GAG-sheddase activated during sepsis-induced ALI/ARDS [38]. Crocin has been reported to inhibit cathepsin L and heparinase, protecting against HS degradation and preserving glycocalyx integrity [39]. Concurrently, recent studies showed that inhibiting angiopoietin-2 (Ang2) could reduce the degree of glycocalyx degradation and protect against lung injury [40]. Importantly, Ang2, operating in a Heparinase-1-dependent manner, has been identified as a potent catalyst for glycocalyx degradation both in vivo and in vitro [41]. Moreover, the Silent information regulator sirtuin 1 (SIRT1)-mediated pathway has been shown to preserve the HS within the endothelial glycocalyx against LPS-induced ALI/ARDS [42–44]. In a mouse model of CLP-induced lung injury, interferon-β was found to restore endothelial glycocalyx damage by modulating the SIRT1/Heparinase-1 pathway, indicating its potential to protect against endothelial damage during sepsis by suppressing Heparinase-1 expression [45]. Several studies have highlighted the significant role of Heparinase-1 in glycocalyx degradation [46, 47], and inhibiting heparinase has been associated with protective effects after the onset of sepsis [37]. Heparinase-2, lacking glucuronidase activity, may potentially inhibit Heparinase-1 [48]. Moreover, a recent report suggests that inhibiting heparinase can ameliorate LPS-induced ALI/ARDS by safeguarding the pulmonary endothelial glycocalyx and promoting its restoration, offering a promising therapeutic target [39, 49, 50]. Several studies have shown that heparinase inhibitors, such as ulinastatin [46] and crocin [39], can reduce the serum levels of HS in LPS-induced ALI/ARDS to protect the integrity of endothelial glycocalyx. Since heparinase has been discovered to play a role in glycocalyx degradation, researchers have begun investigating the interaction between Heparinase-1 and Heparinase-2. However, whether the relative expression levels of Heparinase-1 and Heparinase-2 determine the extent of HS shedding and subsequent glycocalyx degradation remains an area of ongoing exploration [51].

Except for HS degradation by heparinase, various enzymes also degrade other glycocalyx components, such as GAG and PG. HA can be degraded by six different hyaluronidases [52], and evidence has shown elevated levels of HA in septic patients compared to non-septic individuals [53]. Notably, while pathogenic microorganisms are known to produce hyaluronidases [54], the precise mechanisms governing their upregulation in vitro remain incompletely understood. Members of the A Disintegrin and Metalloproteinases (ADAMs) and matrix Metalloproteinase (MMPs) families can cleave PGs from the endothelial glycocalyx, leading to their shedding into the plasma [55, 56]. ADAM family members are upregulated during sepsis, with their levels correlating with disease severity and outcomes. For instance, in pre-clinical sepsis models and ex vivo preparations of human lungs perfused with LPS, ADAM15 could cleave PGs from the endothelial glycocalyx [56]. The concentration of MMPs in the plasma has similarly been shown to correlate with the severity of sepsis, and inhibiting MMPs has been demonstrated to prevent sepsis-induced ALI/ARDS, particularly MMPs-7, MMPs-9, and MMPs-13 [57–60]. The enzymatic degradation of the endothelial glycocalyx carries significant physiological implications, as the degradation products can disseminate through the bloodstream and affect distant sites, thereby influencing the severity of lung injury and prognosis.

### Increased adhesion and recruitment

Pro-adhesion is a phenotypic change in activated ECs that leads to the adhesion of leukocytes to the vessel wall, thereby promoting local inflammatory responses, including the release of inflammatory factors. Activated ECs respond specifically to inflammatory factors secreted by leukocytes by expressing adhesion molecules, such as P-selectin, E-selectin, intercellular cell adhesion molecule-1 (ICAM-1) and vascular cell adhesion molecule-1 (VCAM-1), on their cell surface, which then facilitates the rolling and strong adhesion of leukocytes on the vascular endothelial surface and subsequently promotes the migration of leukocytes into the underlying tissues [61]. LPS can activate monocytes to induce programmed cell death (apoptosis) in ECs through a combination of TNF-α-dependent and TNF-α-independent mechanisms, exacerbating the pro-inflammatory response [62]. In LPS-induced septic ALI/ARDS, pulmonary microvascular ECs are stimulated to release TNF and IL-8, which is accompanied by an increase in intracellular calcium levels. Cytosolic calcium oscillations then induce proinflammatory gene transcription and endothelial E-selectin expression, initiating a series of activated reactions [63]. Subsequently, high mobility group box 1 (HMGB1) can be secreted by ECs after LPS stimulation, leading to increased expression of cytokines, adhesion molecules and chemokines, which further exacerbates inflammation and injury [64]. The degradation of the glycocalyx structure exposes adhesion molecules like E-selectin and ICAM-1 on the denuded endothelium and induces the recruitment of leukocytes, contributing to neutrophil adhesion and leading to diffuse alveolar damage during sepsis-induced ALI [30]. The mRNA and protein levels of Syndecan-4, one of the components of the glycocalyx, are significantly increased following inflammatory injury. Its downregulation severely exacerbates leukocyte adhesion and inflammatory responses in both in vivo and in vitro models of sepsis-induced ALI/ARDS [65]. Research using genetic and pharmacological approaches has revealed that the glycolytic regulator 6-phophofructo-2-kinase/fructose-2, 6-biphosphatase (PFKFB3) can increase the expression of adhesion molecules and promote monocyte adhesion in ECs, which explains why increased glycolysis can worsen pulmonary inflammation and damage during sepsis-induced ALI/ARDS [66]. Furthermore, when ECs interact with epidermal growth factor receptors, they can activate tumor necrosis factor receptor-1 (TNFR1)-mediated inflammation [15]. These findings collectively illustrate that molecules released from leukocytes can regulate ECs, while ECs themselves can express cytokines to recruit leukocytes and facilitate their migration into deep tissues. As leukocytes traverse blood vessels, they become locally activated by chemokines released by ECs, resulting in the expression of integrins on their surface, facilitating firmer adhesion to ICAM-1 and VCAM-1 and initiating their transendothelial migration into injured tissues [67].

The NF-κB pathway is one of the most classical and important inflammatory signaling pathways during sepsis-induced ALI/ARDS. In a resting state, NF-κB, a dimeric transcription factor found in B lymphocytes, is bound to NF-κB inhibitor (IκB) in the cytoplasm. However, when external stimuli are encountered, such as LPS, IκB kinase (IKK) can become activated, leading to the detachment and degradation of the IκB protein from NF-κB. Subsequently, NF-κB is able to bind to specific DNA regions, initiating the transcription of several genes that upregulate the levels of proinflammatory cytokines such as IL-2 and IL-6, which in turn activates the NF-κB signaling cascade in an autocrine manner, amplifying the inflammatory response. Research has demonstrated that, after LPS stimulation, vascular adhesion molecules are highly secreted via the SIRT1/NF-κB/NLRP3 pathway [14]. In sepsis-induced ALI/ARDS, dysregulation of the NF-κB pathway in ECs can result in abnormal chemokine production and the recruitment of massive inflammatory cells, leading to excessive inflammation and tissue damage [68, 69]. In septic mice, the selective blockade of EC-intrinsic NF-κB pathway significantly reduced lung inflammatory injury and mortality and alleviated endothelial dysfunction [70]. Additionally, activation of the NF-κB pathway can stimulate ECs to express adhesion molecules, which increases the binding and detachment of leukocytes and ECs and provides the foundation for subsequent leukocyte transmigration. Moreover, activated ECs release danger-associated molecular patterns, such as histones (particularly H3 and H4), which can further induce the nuclear factor kappa B inflammatory cascade, upregulate EC adhesion molecules such as ICAM1, VCAM1 and E-selectin, and release of inflammatory cytokines at high doses of H3 and H4 [71]. Thus, the NF-κB pathway represents a pivotal and prominent therapeutic target in sepsis-induced ALI/ARDS.

Furthermore, the production of reactive oxygen species (ROS), reactive nitrogen species and other oxidants by activated ECs saturate local antioxidants and contribute to tissue injury directly by downregulating VE-cadherin, upregulating neutrophil adhesion molecule expression and releasing neutrophil chemotactic factors [29]. Targeting NADPH oxidase 4 (NOX4) has been suggested as a potential innovative treatment approach. Sun et al. reported that ECs lack formyl peptide receptors but can be activated by mitochondrial proteins (mt-proteins), suggesting that non-formylated mt-proteins serve as endogenous substances that activate ECs, further increasing EC permeability and promoting adhesion between neutrophils and ECs [72].

### Increased permeability

Increased endothelial permeability, indicating an imbalance of vascular homeostasis due to endothelial barrier dysfunction, is a major pathological feature of sepsis-induced ALI/ARDS. As described above, the glycocalyx is targeted and shed by inflammatory mediators, leading to a thinner glycocalyx layer. Numerous findings have demonstrated that inflammatory stimuli or several inflammatory factors may accelerate the degradation of the endothelial glycocalyx, including TNF-α [37], ROS [73] and others. Moreover, MMPs can directly cleave PGs, including syndecan-1 [35, 74]. Following the degradation of the glycocalyx, mediated by various enzymes and signals, the binding proteins on ECs are reduced to some extent, thereby increasing endothelial permeability, allowing plasma proteins (e.g., albumin) and fluid to move across the vascular wall, resulting in tissue edema formation [35, 75]. Ang-2 is recognized as an intrinsic antagonist of Ang-1 secreted by ECs, which can also mediate glycocalyx degradation [35, 40]. Normally, Ang-2 prevents anti-inflammatory signaling induced by the stable binding of Ang-1 to Tie receptor 2 (Tie2). When activated by Ang-1, Tie2 inhibits the transcriptional activity of the forkhead box protein O1 (FOXO1) transcription factor [76], which further promotes vascular endothelial stability and reduces endothelial glycocalyx degradation through various mechanisms, such as inhibiting Ang-2 production. However, it has also been demonstrated that Tie2 activation promotes the protection and reconstruction of the endothelial glycocalyx in sepsis [77], providing a therapeutic strategy to mediate Ang-2 and Tie2 to protect the endothelial glycocalyx and reduce the increased permeability of endothelial cells [78, 79]. Additionally, the glycocalyx plays a vital role in limiting the interaction between blood leukocytes and the endothelium by “hiding” endothelial cell-associated adhesion molecules, including integrins and immunoglobulin superfamilies. Currently, other glycocalyx fragments, such as HA and HS, have been used as markers of endothelial injury [80]. Thus, there is no doubt that glycocalyx degradation leads to alterations in endothelial permeability, resulting in or exacerbating tissue edema, interstitial fluid shifts, and pulmonary edema, making it a promising target for the treatment of pulmonary endothelium. Nonetheless, the geographic heterogeneity of glycocalyx structure in different vascular locations or at various time points, along with the signaling mechanisms involved in degradation and GAG regulation, remain subjects of current investigation. These areas hold the potential to unveil specific mechanisms.

Furthermore, EC contraction is a known factor that can lead to increased permeability, contributing to thrombosis formation in damaged areas and associated coagulopathy and disorders. One potential mechanism involves the activation of myosin light chain (MLC) kinase (MLCK). Thrombin, a disordered thrombin, cleaves and activates its G-protein-coupled receptor, protease-activated receptor-1 (PAR-1), which triggers the activation of phospholipase C through Gq protein engagement, leading to an increase in intracellular Ca^2+^. Consequently, Ca^2+^/calmodulin (CaM)-dependent MLCK is activated, resulting in the phosphorylation of MLC and subsequent actomyosin interaction, inducing cell contraction [81]. Additionally, Src-mediated tyrosine phosphorylation of the unique N-terminal fragment of EC MLCK can activate EC MLCK. Notably, LPS-induced Rho activation relies on Src family kinases’ activity and direct nitration of RhoA at a tyrosine side chain [82, 83]. Rho, by directly or indirectly increasing MLC phosphorylation, activates the downstream effector Rho-kinase, leading to the accumulation of phosphorylated MLC and EC contraction [84]. Overall, thrombin has been shown to increase EC permeability in a Src/MLCK-dependent manner via an MLC-mediated contractile mechanism [85]. Moreover, histamine and, to a lesser extent, thrombin activates protein kinase C-potentiated phosphatase inhibitor of 17 kD (CPI-17) in a PKC-dependent manner in ECs. The CPI-17-mediated mechanism involves the inhibition of myosin light chain phosphatase (MLCP) in EC barrier regulation, suggesting that artificially induced depletion of CPI-17 can mitigate the increase in microvascular endothelial permeability [86–88]. These studies and observations offer valuable insights for clinical diagnosis and treatment, including potential clinical trials.

Furthermore, increased permeability exposes various sites and receptors on endothelial cells (ECs), leading to their recognition and interaction with various cells, including neutrophils. This interaction triggers EC activation, resulting in the release of leukocytes from the blood vessels. When stimulated by inflammatory mediators, ECs contract, creating gaps between adjacent cells. This phenomenon significantly contributes to increased vascular permeability and may exacerbate inflammatory responses and oxidative stress or weaken the anticoagulant effect. Various junctional proteins organize into two main complexes: TJs and AJs. These complexes not only form the endothelial barrier and regulate paracellular permeability but also provide mechanical stability by linking the plasma membrane of adjacent cells to the actin cytoskeleton. Additionally, several receptor families participate in endothelial barrier function and vascular permeability. Among them, the Tie receptor family, comprising Tie1 and Tie2, is predominantly expressed by ECs. The PARs family and Rho-associated coiled-coil–forming protein kinases (ROCK) family can either disrupt or protect barrier function, depending on the specific activation of intracellular signaling pathways [10, 31, 89]. In summary, various physical injuries, inflammatory mediators, oxidative stress responses, and other factors can damage the pulmonary endothelium and exacerbate lung injury, interfering with endothelial permeability a therapeutic target.

### Coagulant damage

**Fig. 2:**
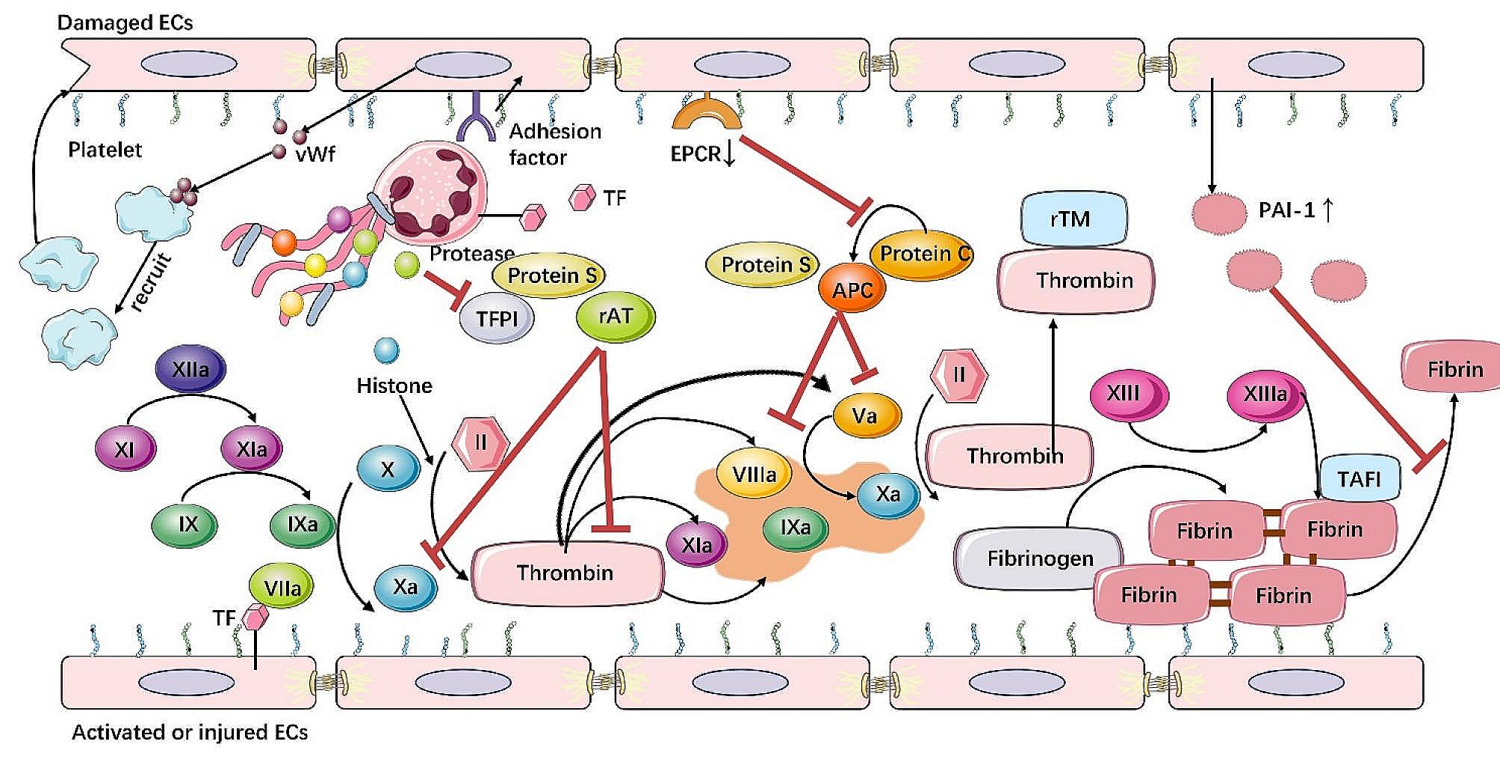
**Mechanism of glycocalyx degradation in LPS or septic conditions. Mechanisms of intravascular coagulation in LPS or septic conditions**. In LPS or septic conditions, anticoagulant and coagulant balance in the intravascular environment can be disrupted by the disruption of endothelial glycocalyx, down-regulation of endothelial thrombomodulin, and decline of plasma anticoagulant proteins such as tissue factor pathway inhibitor (TFPI) and antithrombin. Additionally, activated endothelial cells (ECs) and leukocytes release tissue factor (TF) into the bloodstream, triggering intravascular coagulation. Neutrophil extracellular traps (NETs) also contribute a plethora of proteins that participate in coagulation. In conditions induced by lipopolysaccharide (LPS) or sepsis, fibrinolysis inhibitors such as plasminogen activator inhibitor-1 (PAI-1) and thrombin-activatable fibrinolysis inhibitor (TAFI) are up-regulated, further hindering the fibrinolytic process and potentially leading to disseminated intravascular coagulation (DIC). Recombinant thrombomodulin (rTM) and antithrombin gamma (rAT) represent potential therapeutic agents that could rebalance anticoagulant and coagulant activity in LPS or septic conditions. Moreover, the upregulation of adhesion factors promotes ECs to secrete von Willebrand factor (vWf), which in turn recruits platelets to aid in the repair of damaged ECs. However, shedding of endothelial protein C receptor (EPCR) from ECs results in impaired conversion of protein C to activated protein C (APC), further complicating the coagulation imbalance in these conditions

It is known that multiple mechanisms within the coagulation system act simultaneously to promote a procoagulant state of ECs. In a normal state, the negatively charged GAGs on the endothelial surface prevent platelet adhesion. Healthy ECs inhibit platelet aggregation and fibrin formation [90]. However, when activated, ECs secrete numerous cytokines that enhance platelet adhesion, thereby modifying coagulation function and leading to coagulation disorders. In severe cases, these disorders can progress to disseminated intravascular coagulation (DIC), characterized by abnormal coagulation activation within blood vessels and inadequate coagulation activation outside of blood vessels [91]. Three vital physiological anticoagulant pathways regulate coagulation activation: the tissue factor pathway inhibitor (TFPI), the activated protein C (APC) system, and the antithrombin system. These pathways are notably impaired during sepsis-induced ALI/ARDS [92]. Coupled with disrupted endogenous fibrinolysis, sepsis-induced ALI/ARDS can exacerbate coagulation abnormalities (Fig. 2).

TFPI is located in ECs, megakaryocytes and platelets, and it plays a crucial role in maintaining the balance between coagulation and anticoagulation. TFPI is a Kunitz-type protease inhibitor that directly inhibits the coagulation cascade by targeting free factor Xa and the tissue factor (TF)/factor VIIa/factor Xa complex. TFPI typically exists in three isoforms: α, β, and δ [90]. Under normal conditions, TF binds to factor VIIa, forming the TF/factor VIIa complex, which activates factor X into factor Xa. Factor Xa then combines with factor Va to create the prothrombinase complex on the endothelial surface. Subsequently, the prothrombinase complex converts prothrombin into thrombin, leading to the cleavage of fibrinogen into fibrin [31]. Additionally, Protein S assists TFPI in inhibiting factor Xa activity, thereby causing thrombin synthesis disorders [93]. In septic or LPS conditions, activated ECs and leukocytes generate TF within the blood vessels [94]. Increased TF promotes pathological platelet-vessel wall interactions and microvascular thrombosis. Platelets adhere to ECs, further enhancing endothelial and coagulation activation through various mechanisms, leading to the up-regulation of adhesion molecules and TF expression [89]. Exposed adhesion molecules on the pulmonary endothelium or damaged blood vessels prompt ECs to contribute to hemostasis by producing von Willebrand factor (vWf), either constitutively or in response to chemical or mechanical stimulation from storage granules known as Weibel-Palade bodies [95], which mediates initial platelet adhesion to areas of vascular injury [31]. This vWf can form prothrombotic ultra-large vWf multimers at high levels due to the inactivation of ADAMTS-13(A Disintegrin And Metalloprotease with a ThromboSpondin type 1 motif, member 13) in septic patients [96, 97]. In a murine model of ALI, following LPS administration, TFPI protein expression in lung tissue was significantly decreased while TF expression was increased [98, 99]. Additionally, in TFPI conditional knockout mice, TFPI deficiency worsened sepsis-induced ALI/ARDS and reduced survival rates [98]. Several reports have demonstrated that TFPI can be used in the treatment of Gram-negative bacterial infections, suggesting a therapeutic strategy targeting the coagulation pathway [100–102], and nebulized or injected recombinant human TFPI has been reported to mitigate both pulmonary and systemic coagulation [103, 104]. However, the relationship between the efficacy of exogenous TFPI and the dosage, as well as any associated side effects, remains to be explored.

In addition to the role of TFPI in sepsis-induced ALI/ARDS, the activated protein C (APC) system is also significantly disrupted [105]. TM, expressed in ECs along with thrombin, facilitates the thrombin-catalyzed conversion of protein C to APC [91, 106]. APC limits coagulation amplification by inactivating factors Va and VIIIa with support from cofactor protein S [91]. As an important physiological anticoagulant pathway, impaired APC can also exacerbate coagulation disorders in sepsis. Additionally, in a clinical study involving 77 sepsis patients in the ICU, protein C levels were measured, and severe coagulopathy was found to be associated with the levels of anticoagulant markers, including protein C [107]. Under LPS conditions, a marked downregulation of endothelial protein C receptor (EPCR) resulted in impaired conversion of protein C to APC, as the interactions between EPCR and protein C became compromised [108, 109]. Therefore, regulating EPCR levels in ALI/ARDS may improve prognosis. In a mouse model of CLP, isoorientin reduced the shedding of EPCR on the EC membrane, thus mitigating lung damage following sepsis development [110]. Another study demonstrated that APC diminishes the response to bacterial endotoxin and trauma-related injuries in the plasma of patients with severe sepsis and in animal models of LPS-induced sepsis [111]. As such, these findings suggest that inhibiting protein C or addressing EPCR impairment may hold therapeutic potential and reduce the risk of coagulation disorders. However, further research is required to gain a deeper understanding of the underlying mechanisms involving protein C, APC, and EPCR in ECs as a treatment approach for sepsis. Interestingly, in a clinical trial of APC for the treatment of acute lung injury, the results suggested that APC did not improve outcomes from ALI [112], which contradicts findings indicating that infusion of recombinant APC has a beneficial effect on survival in an animal model of ALI induced by sepsis [113].

It is considered that the endogenous coagulation pathway is activated mainly because of the destruction of the endothelial glycocalyx, the downregulation of endothelial TM, the decrease in plasma anticoagulant proteins, and the presence of neutrophil extracellular traps (NETs) [114]. This activation promotes the generation of thrombin and initiates blood clot formation within blood vessels. Due to secondary platelet activation resulting from continuous thrombin formation, a significant number of platelets are consumed while interacting with the endothelial surface, leading to platelet exhaustion and prolonged clotting times in 15 to 30% of septic patients [108]. In clinical treatment, the use of anticoagulants can significantly reduce the risk of thrombosis in small blood vessels in lung injuries. Furthermore, when sepsis induces ALI, the expression of fibrinolytic inhibitors, including plasminogen activator inhibitor-1 [115], is up-regulated, which further impedes the dissolution and clearance of fibrin, resulting in the formation of microvascular thrombosis. Recombinant antithrombin (rAT), as an alternative to plasma-derived antithrombin, can trap activated coagulation factors, including thrombin and factor Xa, within the septic microcirculation. Therefore, rAT could be a therapeutic agent that can restore anticoagulant potential [91, 116]. During fibrinolysis, thrombolytic agents like tissue-type plasminogen activator (t-PA), mainly produced in ECs, and urokinase-type plasminogen activator (u-PA) catalyze the degradation of fibrin within clots. Evidence from cultured ECs, experimental animal models, and sepsis patients suggests that decreased levels of t-PA and u-PA exacerbate lung injury and disrupt the hemostatic balance [117–119]. Thus, coagulopathy is also considered a potential characteristic of endothelial injury. Despite abundant evidence demonstrating the interaction between the coagulation system and inflammatory response, the systematic interaction between the components of the coagulation system and inflammatory response remains unclear.

In addition to that, ECs also interact with platelets, contributing to the pathogenesis of sepsis-induced ALI/ARDS. In this condition, endothelial-derived ADP may trigger platelet activation, following which receptors such as αIIbβ3 integrins [120], CD40L (CD154) and P-selectin become highly expressed on the surface of ECs [121]. These are important mediators in interactions with fibrinogen and other circulating cells in the blood [122]. As previously mentioned, vWf is released from the endothelial Weibel-Palade bodies into the bloodstream. In vessels with lower shear stress and the presence of vWf, erythrocytes tend to aggregate, rolling on and adhering to ultralarge vWf multimer strands released from activated ECs [122]. Subsequently, platelets interact with the A1 domain of vWf, resulting in additional platelet binding and platelet activation [123]. Moreover, various cells in the bloodstream adhere to ECs with the assistance of vWf from platelets. Neutrophils and monocytes in vessels begin to roll over ultralarge vWf multimer strands independently, with neutrophils utilizing neutrophil antigen 3a and monocytes employing P-selectin glycoprotein ligand-1 (PSGL-1) for adhesion [120, 124]. Notably, natural killer (NK) cells in the blood have been observed to adhere to vWf-coated surfaces under flow, likely through a platelet-dependent mechanism [125].

Overall, these interactions involve various blood cells in the bloodstream through EC surface receptors, platelets, and vWf, contributing to EC damage, exacerbation of inflammation or thrombosis, and aiding in the removal of metabolic waste.

### Vasomotor function and angiogenesis function

Vasomotor tone regulation involves a complex interplay of endothelial-dependent and endothelial-independent factors, with the EC phenotype playing a pivotal role. In sepsis, vascular tone dysregulation primarily stems from disruptions in the production of nitric oxide (NO), prostacyclin (PGI), and endothelin [126].

Endothelin-1 (ET-1), a potent vasoconstrictive peptide released by ECs, exhibits a significant increase in release during sepsis-induced ALI/ARDS following EC activation [127]. In murine sepsis models, sitaxentan, a highly selective ET-1 receptor A inhibitor, effectively prevented pulmonary inflammation and fibrosis. Elevated plasma ET-1 levels have been observed in ARDS patients, correlating with aberrant pulmonary ET-1 metabolism, which tends to normalize in recovering patients [128]. Clinical research has also shown that reducing LPS-induced ET-1 levels can protect the diastolic function of pulmonary vessels [129]. Thus, targeting ET-1 could be a potential strategy to improve endothelial dysfunction [130].

PGI belongs to the eicosanoid group of biologically active lipid compounds, which includes primary prostaglandins (e.g., PGE2, F2 α, and D2) as well as PGI2 [131]. A previous study indicated that the barrier-protective effects of PGE2 and PGI on pulmonary ECs are mediated through the PKA and Epac/Rap pathways. These mechanisms are believed to underlie the protective effects of prostaglandins against vascular barrier dysfunction induced by agonists in vitro and against lung injury caused by mechanical stress in vivo [132].

Animal models of sepsis often display a notable surge in nitric oxide (NO) levels during the initial hours of sepsis, primarily attributed to heightened expression of tissue-inducible nitric oxide synthase (iNOS) [133]. Notably, NO generated by iNOS can enhance vasoconstriction by increasing the levels of endothelin-1 (ET-1) and thromboxane A2, effectively inducing vasoconstriction [134]. However, the challenges inherent in measuring NO levels and the dynamic nature of this process pose difficulties in drawing definitive conclusions. Furthermore, insights from animal studies suggest that elevated bioavailability of NO stemming from iNOS may exacerbate lung injury [135]. Several studies have illustrated the involvement of both iNOS and endothelial nitric oxide synthase (eNOS) in stimulating pulmonary endothelial cells to produce significant amounts of NO, resulting in vasoconstriction and an escalation in lung injury [136, 137].

The vascular endothelium, known for its remarkable plasticity, has the potential for vascular regeneration at sites of detachment or rupture of ECs when necessary [138]. It adapts to various functions influenced by different tissues’ specific needs, energy requirements, and unique conditions 139]. Numerous molecules have been identified to be involved in angiogenesis, a process essential for the recovery of lung diseases and the healing of lung injuries [140]. Therefore, mediating, interfering with, or upregulating angiogenesis carries significant implications for improving prognosis. Sphingosine 1-phosphate (S1P) [141], a bioactive metabolite of sphingomyelin, initiates various signaling cascades by binding to its receptors (S1PR1-3) on the surface of ECs. S1P primarily stimulates EC proliferation, survival, migration, and the formation of capillary lumens through its interaction with S1PR1. Additionally, S1P modulates angiogenesis by targeting the transcription factor peroxisome proliferator-activated receptor γ (PPARγ) and forming the S1P/PPARγ/PGC1β complex in ECs [142, 143]. Another crucial factor in angiogenesis is vascular endothelial growth factor (VEGF), which primarily targets ECs and is essential for vasculogenesis and angiogenesis [144, 145]. Reduced production of VEGF in ALI/ARDS may contribute to vascular lesions, as VEGF plays a pivotal role in promoting endothelial survival by inhibiting apoptosis [146]. Administration of VEGF within the vascular system has been shown to regulate the formation of new blood vessels, presenting a promising avenue for sepsis treatment targeting ECs [147, 148]. Studies have indicated that members of the BMP family regulate VEGFR2 and Notch signaling pathways and act through the TAZ-Hippo signaling pathway to fine-tune angiogenesis [149]. A recent study employing genetic lineage tracing and FACS analysis demonstrated that reactivating FoxM1-dependent EC regeneration in ALI mice effectively improved vascular repair, inflammation resolution, and survival in elderly sepsis-induced ALI mice. This finding aligns with results observed in elderly patients with ARDS [150].

Recent early-phase clinical trials have explored the potential of stem cell-based therapies for treating sepsis-induced ALI/ARDS. The endogenous repair mechanism for damaged vascular endothelium relies on the proliferation of local ECs. However, the processes of re-endothelialization and angiogenesis following endothelial injury are also influenced by bone marrow-derived endothelial progenitor cells (EPCs) [151]. Studies have shown that administering EPCs in preclinical sepsis models can lead to beneficial effects such as improved vascularity, organ function, and reduced mortality [152, 153]. Exosomes derived from EPCs contain an abundance of microRNAs-126-3p (miR-126-3p) and miR-126-5p, whose expression is increased in lung tissue when treated with these exosomes. Both miR-126-3p and miR-126-5p target genes associated with the regulation of endothelial activation and inflammation, such as VCAM1 and HMGB1 [154, 155]. Exosomes deliver miR-126-3p and miR-126-5p to ECs, reducing the LPS-induced up-regulation of VCAM1 and HMGB1. Furthermore, miR-126, through exosome-mediated targeting of Sprouty-related EVH1 Domain 1 (SPRED1) and phosphoinositide 3-kinase regulatory subunit 2 (PIK3R2), regulates the endothelial response to VEGF and its role in endothelial permeability and proliferation. As a result, miR-126-3p and miR-126-5p secreted by EPCs contribute to vascular endothelial vasculogenesis, prevent microvascular dysfunction, and potentially improve sepsis outcomes [155]. Therefore, miR-126 secreted by EPCs inhibits various targets that play critical roles in sepsis-induced ALI/ARDS response pathways, including leukocyte trafficking, permeability, and cytokine-mediated inflammation. These findings provide compelling evidence supporting the concept that EPC exosomes may offer therapeutic benefits in sepsis-induced ALI/ARDS through the transfer of miRNAs, and promoting angiogenesis may also contribute to the prognosis of sepsis-induced ALI/ARDS to a certain extent and has shown promise in clinical research [156].

### Imbalance of oxidative stress

Oxidative stress is known to play a significant role in the progression of sepsis-induced ALI/ARDS [11, 157]. Under normal physiological conditions, ROS are essential for various cellular functions, including cell signaling, post-translational protein processing, host defense, gene expression regulation, and cell differentiation. However, excessive ROS production can result in endothelial dysfunction and EC death. The dysfunction and death of pulmonary vascular ECs can result in increased vascular permeability and even vascular rupture. The NOX family of proteins is the primary enzymatic source of ROS, and within ECs, four NOX isoforms are expressed, namely NOX1, NOX2, NOX4 and NOX5 [158]. The activation of NOX leads to EC dysfunction by generating ROS, including superoxide, hydroxyl radicals, and peroxynitrite [159]. Jiang J. et al. demonstrated that NOX4 (NADPH oxidase) activation via the CaMKII ERK1/2 / MLCK pathway plays a pivotal role in REDOX-sensitive activation of ECs in CLP mice [159]. Importantly, p22phox, the only membrane-bound subunit, was found to be essential for the stability and activation of NOX1, NOX2, and NOX4 [11, 158]. Thus, an imbalance of p22phox beyond the self-regulation range corresponds to changes in oxidative stress downstream of the NOX family. In the LPS-induced ALI mouse model, LPS promotes NOX2-mediated ROS production in pulmonary vascular ECs of mice by interacting with TLR4. Additionally, ROS can induce various forms of programmed cell death in ECs, such as pyroptosis, parthanatos, and ferroptosis. Notably, ROS serve as upstream signals for the activation of the NLRP3 inflammasome, which upregulates the expression of NLRP3, pro-caspase-1, and pro-IL-1β, thereby promoting the assembly and activation of the NLRP3 inflammasome [160, 161]. Excessive accumulation of ROS can cause DNA single- and double-strand breaks, leading to overactivation of poly (ADP-ribose) polymerase 1 (PARP-1) and accumulation of poly (ADP-ribose) (PAR), depleting substantial amounts of NAD^+^ [162]. Furthermore, the translocation of PAR from the nucleus to the mitochondria induces the release of apoptosis-inducing factor (AIF) from the mitochondria, forming a complex with macrophage migration inhibitory factor (MIF) in the cytoplasm [163]. This process, including nuclear translocation of the AIF/MIF complex, leads to chromatin condensation and DNA fragmentation, ultimately resulting in EC death [164].

**Fig. 3:**
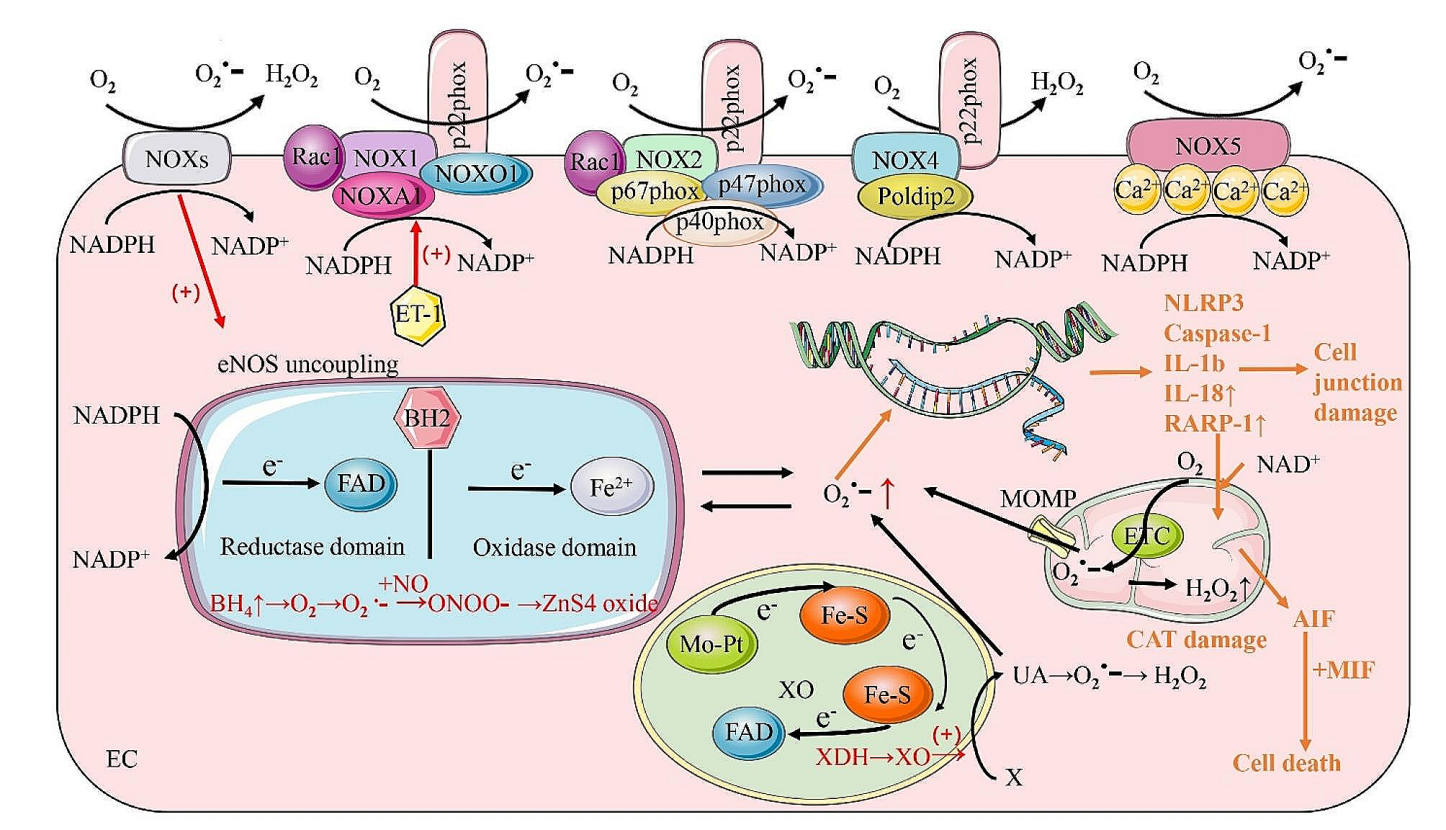
**Oxidative stress response in ECs.** Reactive oxygen species (ROS) within ECs primarily originate from mitochondria, NADPH oxidases (NOXs), endothelial nitric oxide synthase (eNOS) uncoupling, and xanthine oxidase (XO). The generation of ROS through various pathways results in the upregulation of ROS expression in ECs, leading to mechanical cell death and disruption of intercellular junctions

During ALI, various oxidases are activated through different pathways, contributing to oxidative stress. These oxidases include NO synthase (NOS) [165], Xanthine oxidase (XO) [166] and Cytochrome P450 (CYP) [167]. Since many oxidases are present in endothelial mitochondria, and mitochondria are the primary site of REDOX reactions, oxidative stress can lead to mitochondrial damage. Several pathways are involved in this process. For instance, eNOS produces NO (eNO), which can react with O^2−^ to form highly reactive peroxynitrite (ONOO^−^). This increased oxidative and nitrosative stress can activate the nitroprotein RhoA and induce the uncoupling and translocation of eNOS to mitochondria, leading to endothelial barrier dysfunction and lung injury [137, 168]. Nuclear factor erythroid 2-related factor 2 (Nrf2), possessing antioxidative potential, is a transcription factor that interacts with multiple signaling pathways and regulates the activity of various oxidases (NOX, NOS, XO, and CYP) associated with inflammation and apoptosis [169]. Nrf2 plays a pivotal role in ALI by exerting antioxidant and anti-inflammatory functions. Marika et al. investigated the potential of cashew nuts, a prominent source of polyphenols in the global diet, to alleviate sepsis-induced ALI/ARDS through the Nrf2 signaling pathway [170]. Additionally, Hong et al. demonstrated that Hydnocarpin D attenuates LPS-induced ALI via Nrf2-associated pathways, indicating that the Nrf2-associated pathway may inhibit oxidative stress and the inflammatory response [171]. Similarly, Lv et al. showed that xanthohumol markedly attenuated the oxidative stress response and ameliorated LPS-induced ALI in mice by inducing the AMPK/GSK3beta-Nrf2 signaling axis in vivo [172]. Both mitochondrial damage and nuclear chromosomal damage in ECs, as well as alterations in enzyme levels within ECs, can result in varying degrees of EC dysfunction, which can disrupt normal mitochondrial oxidative metabolism functions and lead to severe EC damage and detachment (Fig. 3).

## Interaction between endothelial cells and immune cells in sepsis-induced ALI/ARDS

During the progression of sepsis-induced ALI/ARDS, there are concurrent and interwoven proinflammatory and anti-inflammatory responses. A pivotal aspect of sepsis-induced ALI/ARDS lies in the interaction between ECs and various inflammatory cells, while cytokines and inflammatory factors play indispensable roles in this cascade. This interaction between cells and effector molecules stands as the primary pathophysiological alteration in sepsis-induced ALI/ARDS [173]. The inflammation observed in sepsis-induced ALI/ARDS can be initiated through both exogenous and endogenous pathways. Exogenously, pathways activated by LPS trigger inflammatory responses by engaging Toll-like receptors (TLRs). The LPS-mediated TLR4 and caspase-11 (or human caspase-4/5) cascade can elevate the production of proinflammatory/anti-inflammatory mediators, induce pyroptotic cell death and lead to immune dysfunction. Conversely, endogenous pathways primarily involve danger signal molecules, known as damage-associated molecular patterns (DAMPs), which are released by local inflammatory cells or dying cells. These DAMPs recruit and activate immune cells by binding to various receptors, including IL-6, IL-10, and IL-33, among others [174]. As previously mentioned, disruption of the endothelial barrier leads to excessive leakage of protein-rich fluid, diverse blood cells, and inflammatory cells into the interstitium and alveoli. Consequently, white blood cells migrate along ECs, triggering neutrophil activation, which, along with macrophages and various inflammatory cells, releases a plethora of substances within the lung, thereby exacerbating inflammation. Furthermore, these interactions can serve as biomarkers for sepsis-induced ALI/ARDS [175, 176] (Table 1), including intricate interactions between ECs and various cell types, representing a focal point of research efforts. Thus, targeting these interactions holds promise for the treatment or amelioration of sepsis-induced ALI/ARDS.

**Table 1:** Biomarkers of sepsis-induced ALI/ARDS

Biomarkers	Detection object	Change	Reference
Receptor for advanced glycation end products (RAGE)	Plasma and alveolar fluid clearance	↑	[177]
Specific surfactant proteins (SP)	Pulmonary edema fluid; plasma	SP-D↓; SP-A↑	[178]
Membrane glycoprotein KL6	BALF and plasma	↑	[179]
Club cell secretory protein (CCSP)	Plasma	↑	[180, 181]
Soluble intercellular adhesion molecule-1 (sICAM-1)	Plasma and edema fluid	↑	[182]
Angiopoietin1 (Ang-1) and angiopoietin-2 (Ang-2)	Plasma	↑	[183–185]
E-selectin	Plasma	↑	[186]
IL-1β, TNFα, IL-8 and IL-6	Plasma	↑	[187, 188]
IL-10	Plasma	↓	[188]
High mobility group box nuclear protein (HMGB) 1	Plasma	↑	[189]
Lipopolysaccharide-binding protein (LBP)	Plasma	↑	[190]
Plasminogen activator inhibitor (PAI-1)	BALF	↑	[191]
Thrombomodulin (TM)	Pulmonary edema fluid and Plasma	↑	[192]
Protein C	Plasma	↓	[193]
Keratinocyte growth factor (KGF) and hepatocyte growth factor (HGF)	BALF	↓	[194, 195]
Vascular endothelial growth factor(VEGF)	Epithelial lining fluid	↑	[196]
KL-6	Plasma	↑	[197, 198]

### Interaction between endothelial cells and leukocytes

In ALI, various immune cells, including lymphocytes and macrophages, initiate a potent inflammatory response that exacerbates lung injury upon interaction with ECs. This interplay between the endothelium and leukocytes is a frequent occurrence in ALI. Leukocyte-generated thrombin can activate PARs found on both ECs and leukocytes, prompting ECs to release inflammatory factors such as IL-6. Thrombin’s effect on ECs increases the expression of selectin E and P on the EC surface, thereby augmenting leukocyte adhesion to ECs and facilitating leukocyte exudation and chemotaxis [199]. Conversely, inflammatory factors and chemokines can attract a substantial number of inflammatory cells to accumulate at the site of inflammation, which produce numerous cytokines within the inflamed area, culminating in a cytokine storm.

### Interaction of ECs with neutrophil

Neutrophils have long been recognized for their pivotal role as immune effector cells in the pathogenesis, progression, and resolution of various diseases, including ALI/ARDS [200]. Recent research has elucidated the significant contribution of extracellular histones in promoting neutrophil adhesion and subsequent activation. This cascade begins with histones stimulating the pulmonary endothelium via TLR signaling, leading to P-selectin translocation and vWf release [201]. Concurrently, intracellular histones are released into the extracellular space, where they serve as inflammatory mediators in cells, tissues, and organs.

**Fig. 4:**
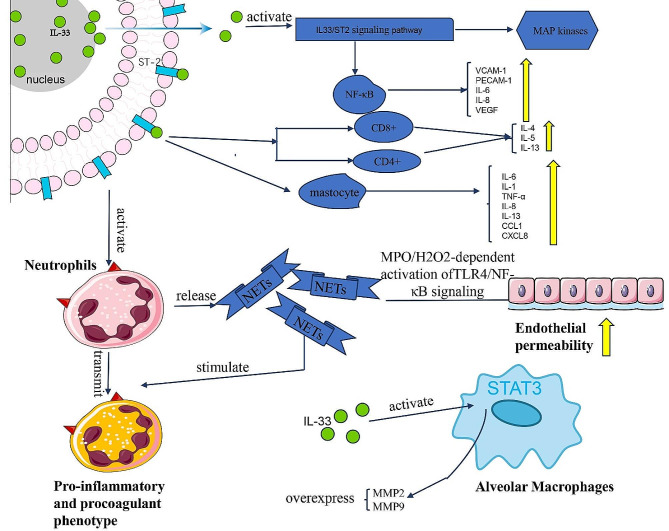
**The cascade of effects resulting from the release of IL-33 under the action of endothelial injury and other factors.** Initially expressed within the nucleus, endogenous IL-33 expression is upregulated by endothelial injury and other stimuli, leading to the release of a significant amount of IL-33 outside the cell. Extracellular IL-33 binds to its receptor ST2, initiating downstream signaling events such as NF-κB and MAP kinase activation. Damaged endothelial cells promote the transition of neutrophils into pro-inflammatory and pro-coagulant phenotypes. Neutrophils release neutrophil extracellular traps (NETs), which further exacerbate neutrophil phenotypic transformation and enhance endothelial permeability through the MPO/H_2_O_2_-dependent activation of the TLR4/NF-κB signaling pathway. IL-33 also acts on CD8^+^ T cells and CD4^+^ T cells, leading to increased expression of IL-4, IL-5, and IL-13. Additionally, IL-33 stimulates mast cells, resulting in increased expression of IL-6, IL-1, IL-8, IL-13, CCL1, CXCL8, and TNF-α. Furthermore, IL-33 induces overexpression of MMP2 and MMP9 in macrophages

IL-33, recently discovered to be expressed in ECs, epithelial cells, and fibroblasts, plays a pivotal role in mediating crucial interactions (Fig. 4) [202]. IL-33 targets various immune cells, including eosinophils, mast cells, and macrophages. Both isoforms of IL-33, namely proIL-33 and mtrill-33, serve as immune adjuvants capable of eliciting substantial Th1 CD4^+^ and CD8^+^ T cell responses. This stimulation results in the production of Th2-related cytokines, specifically IL-4, IL-5, and IL-13, leading to histopathological alterations in the lungs. IL-33 also induces the generation of proinflammatory cytokines and chemokines (such as IL-6, IL-1β, TNF-α, IL-8, IL-13, CCL1, and CXCL8) by human mast cells and cooperates with IgE to enhance cytokine production [203, 204]. Its receptor, ST2, encodes a soluble secretory ST2 (sST2), which functions as a component in IL-33 signaling [205]. Human basophils and ECs express ST2 receptors at high levels and respond to IL-33 by producing increased levels of IL-1β, IL-4, IL-5, IL-6, IL-8, IL-13, and granulocyte-macrophage colony-stimulating factor (GM-CSF) [206, 207]. IL-33 stimulates the production of nitric oxide (NO) in ECs through the ST2/TRAF6-Akt-eNOS signaling pathway, thereby promoting angiogenesis and increasing vascular permeability [208]. Thus, the role of cell-mediated interactions facilitated by cytokines cannot be underestimated. In normal physiological conditions, endogenous IL-33 is consistently expressed within the nucleus, typically binding to chromatin by interacting with histone H2A/H2B. However, when tissue damage, mechanical stress (such as necroptosis or cellular stress), or endothelial injury, including damage or detachment, occurs, the expression of IL-33 is upregulated. Consequently, a significant amount of IL-33 is released into the extracellular space [209]. Subsequently, IL-33 in ECs binds to ST2, which is widely distributed on the surfaces of ECs and inflammatory cells, initiating the IL-33/ST2 signaling pathway [210]. The binding of IL-33 to ST2 triggers downstream signaling events, including the activation of NF-κB and MAP kinases (ERK, p38, and JNK). Activation of the NF-κB signaling pathway amplifies the pro-inflammatory and pro-angiogenic responses of ECs by elevating the expression of adhesion molecules like VCAM-1, platelet EC adhesion molecule-1, and the secretion of cytokines such as IL-6, IL-8, and VEGF [92].

As mentioned above, ECs can activate neutrophils, indirectly leading to an increase in the release of NETs, which can enhance endothelial permeability and, conversely, promote neutrophil transformation into pro-inflammatory and procoagulant phenotypes, indicating that neutrophils and NETs promote the pro-inflammatory and pro-angiogenic processes of ECs, further exacerbating immune system dysfunction. Wojciak-Stothard et al. demonstrated that NETs could induce pro-inflammatory and pro-angiogenic responses in human pulmonary artery ECs via MPO/H_2_O_2_-dependent activation of the Toll-like receptor 4 (TLR4)/NF-κB signaling pathway [211]. To reduce NETs-mediated lung damage and inflammation, DNase is commonly used in preclinical ALI models due to its capacity to degrade the NET DNA scaffold. However, it is noteworthy that some research has indicated that DNase treatment may lead to increased systemic bacterial burden and reduced survival rates [212]. In a study conducted by Lafrançais et al., higher levels of NETs were observed in ARDS patients with infectious origins, and these levels were correlated with worse clinical outcomes. Furthermore, their research revealed that NETs release exacerbated ALI symptoms, but this effect could be alleviated through the administration of DNase. Consequently, DNase presents a potential target for disrupting NET-mediated interactions between ECs and neutrophils [213]. However, to safely leverage these observations, further investigation is required to elucidate the pathways that regulate the equilibrium between neutrophil activation and desensitization.

### Interaction of ECs with macrophages

At various pathological stages, macrophages undergo phenotypic changes regulated by factors such as suppressor of cytokine signaling (SOCS) 1/SOCS3 and interferon regulatory factor (IRF) 4/IRF5, resulting in distinct functional roles [214]. Initially, macrophages exhibit a predominantly pro-inflammatory M1 phenotype, which is involved in defense and marked by the release of numerous pro-inflammatory mediators. However, as the disease progresses, macrophages transition towards the anti-inflammatory M2 phenotype, participating in tissue remodeling and potentially exacerbating tissue fibrosis to some extent [214, 215]. A study published in 2008 reported that alveolar macrophages (AMs) could directly enhance the pulmonary microvascular endothelium through iNOS [216]. Recently, research has demonstrated that the regulator of G protein signaling-1 (RGS1), a key member of the RGS family [217], co-regulates the immunophenotype of the AMs subpopulation through PLC-IP3R signal-dependent intracellular Ca^2+^ responses [218]. Moreover, evidence confirms that M2 macrophages release anti-inflammatory and pro-growth cytokines, both in vitro and in vivo, to accelerate the proliferation of lung ECs and improve survival in mice with sepsis-induced ALI/ARDS [219]. Pathologically, elevated IL-33 activates signal transducers and activators of transcription 3 (STAT3) in AMs, leading to increased expression of MMP2 and MMP9, which further damages alveolar ECs and exacerbates the disease [220]. Additionally, the VEGF-C/VEGFR-3 signaling in macrophages contributes to ameliorating ALI/ARDS through multiple functions, including increased production of anti-inflammatory cytokines and enhanced efferocytosis [221]. Nonetheless, the mechanisms governing the interaction between macrophages and ECs remain unclear, and further research is needed to elucidate how various macrophage phenotypes interact with ECs at different locations within the lung. Nevertheless, regulating the function of macrophages holds promise as a therapeutic strategy against ALI/ARDS.

## Interaction between endothelial cells and stromal cells in sepsis-induced ALI/ARDS

During the pathogenesis of ALI and ARDS, there is ongoing interaction between pulmonary capillary endothelium and lung stromal cells, including fibroblasts and epithelial cells. Notably, the interplay between lung epithelial cells and ECs significantly impacts the disease progression. These interactions not only influence the phenotype of ECs but also affect the differentiation and secretion function of other cell types.

### The interaction between endothelial cells and alveolar epithelial cells

Alveolar capillary ECs are intimately associated with alveolar epithelial cells, making epithelial-endothelial crosstalk crucial in sepsis-induced ALI/ARDS. Damage to the alveolar epithelial-endothelial barrier, where gas exchange occurs in the lung, leads to the accumulation of proteinaceous fluid filled with proteins and cells in the alveolar space. This disrupts alveolar gas exchange, resulting in severe lung dysfunction [222–224]. Pathological specimens from ALI/ARDS patients often reveal diffuse alveolar damage characterized by alterations in endothelial and epithelial cells [225]. Pulmonary fibrosis is a common complication of primary pulmonary ALI/ARDS [20]. Its pathogenesis has transitioned from being driven by fibroblasts to being governed by epithelial cells, involving intricate crosstalk among alveolar epithelial cells, fibroblasts, immune cells, and ECs [226]. Single-cell RNA sequencing data has confirmed that alveolar epithelial cells serve as the source of fibroblasts and myofibroblasts in idiopathic pulmonary fibrosis. Dysregulated epithelial cells interact with ECs through various signaling mechanisms, activating fibroblasts and myofibroblasts. Additionally, alveolar epithelial cells secrete senescence-associated secretory phenotypes, further promoting fibrosis [227]. Wang et al. demonstrated that alveolar epithelial cells protect ECs from septic hyperpermeability by secreting a variety of anti-inflammatory and antimicrobial factors [228]. Furthermore, alveolar epithelial cells contribute to the pathology of sepsis-induced ALI through ferroptosis induced by neutrophil extracellular traps (NETs), exacerbating damage to the alveolar endothelium [229]. The interaction between epithelial cells and ECs is notable when activated by IL-33, resulting in increased production of IL-6 and IL-8 [230], which upregulates HIF-1α and VEGF expression in vascular ECs [231], causing additional damage to the endothelium and worsening ALI/ARDS. Hence, the scientific and potential clinical therapeutic importance of epithelial-endothelial crosstalk in maintaining alveolar integrity in ALI/ARDS is evident. Future studies will further define the soluble factor(s) responsible for pulmonary EC protection and explore the therapeutic potential of this epithelial-endothelial interaction.

### The interaction between endothelial cells and fibroblasts

In the progression of pulmonary fibrosis in ALI/ARDS, fibroblasts can undergo activation into myofibroblasts, which persist in cases of fibrosis [232]. Besides endogenous tissue fibroblasts, myofibroblasts can also originate from ECs through endothelial-mesenchymal transition [233]. A significant aspect of the initial injury in pulmonary fibrosis involves the creation of a profibrotic environment due to repetitive micro-injuries. Within this milieu, various factors, including cytokines, chemokines, and growth factors, coordinate the recruitment of fibroblasts, contributing to fibrosis and lung injury [234, 235]. Notably, VEGF-A, which is abundantly secreted by ECs, alveolar epithelial cells, and B cells in the lung, plays a pivotal role in maintaining alveolar integrity. Recent evidence suggests that VEGF-A can induce the migration and activation of fibroblasts, thereby contributing to pulmonary fibrosis [235]. However, conflicting evidence exists regarding the role of VEGF-A in pulmonary fibrosis, with some studies indicating its protective role when secreted by type II alveolar epithelial cells [236, 237]. The specific mechanism of VEGF-A in the progression of fibrosis and ALI/ARDS as a whole remains to be further confirmed. In addition, scRNA-seq data from rats with pulmonary fibrosis have highlighted the significant role of ECs in stimulating fibroblast proliferation [238]. Pulmonary ECs are also known to secrete fibroblast growth factor (FGF) [239], which has been shown to promote the division and proliferation of fibroblasts. FGFs are crucial for the development and repair of lung tissue following ALI/ARDS [239]. In a mouse model of LPS-induced ALI, FGF1 has been found to effectively reduce inflammation and oxidative stress during lung injury, exerting a protective role [240]. Similarly, FGF4 has shown protective effects against LPS-induced lung injury both in vivo and in vitro, as evidenced by reduced lung tissue damage, apoptosis, and inflammation following treatment with recombinant FGF4 [241]. Additionally, FGF10 has been demonstrated to play a protective role in LPS-induced ALI by increasing the population of mesenchymal stem cells (MSCs) [242]. Notably, certain basal cells, including fibroblasts, rely on FGF-associated signaling for their survival, proliferation, and differentiation, presenting potential therapeutic targets for lung repair [243]. However, further research is needed to confirm the effects of these findings in clinical settings. Moreover, vascular and pulmonary connective tissue growth factor (CTGF) induces fibroblast differentiation and promotes pulmonary fibrosis through various cell signaling pathways, such as integrin-dependent pathways [244].

Thus, the interaction between ECs and lung stromal cells plays a crucial role in shaping the progression of the entire disease process [245]. However, the molecular mechanisms underlying this interaction remain unclear. Therefore, gaining a detailed understanding of the intercellular crosstalk between ECs and lung stromal cells holds great significance for advancing treatment strategies.

## The endothelium as a therapeutic target in sepsis-induced ALI/ARDS

While preclinical studies in animal models of ALI have been conducted for decades, translating these findings into effective treatments or targeted drug therapies for human ARDS remains challenging. However, the advent of single-cell omics technology [246, 247] has facilitated the identification of numerous biomarkers for the prognosis of ARDS in humans (Table 1). These biomarkers offer valuable tools for diagnosing the condition and assessing its prognosis, thereby enhancing our ability to manage the disease effectively.

EC dysfunction encompasses a multitude of signaling pathways regulated by diverse intracellular and extracellular molecules, including second messengers [248]. Thus, elucidating the molecular mechanisms underlying these pathways in sepsis-induced ALI/ARDS holds promise for identifying novel therapeutic targets and insights into clinical management, with the potential to modify disease progression. Notably, the NF-κB pathway serves as a prominent example [67, 69], modulating adhesion, permeability, and inflammatory responses. Consequently, targeting the NF-κB pathway holds significant potential for alleviating the diverse symptoms associated with sepsis-induced ALI/ARDS. It is worth noting that the impact of NF-κB pathway inhibition can vary depending on the stage of disease progression. While NF-κB inhibitors may exert a protective effect when administered before the peak of injury in ALI animal models, their effects can differ during the regression phase or late progression of the disease. In these later stages, NF-κB inhibition may exacerbate endothelial barrier damage, increase endothelial cell apoptosis, and potentially delay tissue repair [240, 249–252]. Millar et al. have extensively reviewed this dual role of targeting the NF-κB pathway in ARDS treatment [70], providing comprehensive insights into this complex topic. Therefore, it is essential to consider the timing of NF-κB inhibition in the context of disease progression to optimize therapeutic outcomes. Additionally, the MAPK pathway plays a significant role in sepsis-induced ALI/ARDS [50], influencing processes such as endothelial cell proliferation, growth, and apoptosis. Interaction between the NF-κB/MAPK-mediated signaling pathway, EC-epidermal growth factor receptor (EGFR), tumor necrosis factor receptor 1 (TNFR1)-mediated inflammation, and receptor-interacting protein 3 (RIP 3)-dependent necroptosis regulation has been demonstrated in ALI [15]. Recent studies have shown that intervention with Yupingfengsan exhibited therapeutic effects on LPS-induced ALI mice by inhibiting the activation of NLRP3 inflammasome and the MAPK signaling pathway [253], which highlights the potential of drugs targeting MAPK in ARDS treatment, underscoring the significance of these signaling pathways in the pathogenesis of ALI/ARDS. Moreover, the Notch pathway has emerged as another common player in ALI/ARDS, influencing various immune and non-immune cells by regulating cell proliferation. Activation of the Notch signaling pathway can promote inflammation through classically activated macrophages (M1), while its inhibition can suppress inflammation by activating alternatively activated macrophages (M2) [254]. In septic ALI/ARDS, the Notch pathway may modulate inflammatory responses through macrophages, activate Dcreg to inhibit inflammation and contribute to pulmonary development, offering potential therapeutic strategies for ALI/ARDS treatment [255]. An increasing number of drugs targeting these signaling pathways have been developed to improve sepsis-induced ALI/ARDS [70, 256, 257]. Dis et al. have summarized the basic signaling pathways of sepsis-induced ALI/ARDS and discussed treatment strategies [68]. However, genetic diagnosis and omics research of human ARDS are still in the early stages. It is hoped that future research will elucidate the interplay between proteins, genes, and signaling pathways, leading to the development of specific treatments. Therefore, deeper research into understanding these typical signaling pathways could be crucial for comprehending and predicting ALI/ARDS.

The identification of targets within typical cell signaling pathways for treatment has shown promising results in improving patient outcomes. Additionally, the emergence of MSCs and their exosomes, as mentioned previously, offers a broader solution to address the current treatment dilemma. Liang et al. made a detailed summary of the use of MSCs and their exosomes for intervention in the treatment of ALI/ARDS [258]. Liang et al. highlighted that MSCs exert their therapeutic effects through paracrine mechanisms, leading to increased expression of anti-inflammatory factors and reduction of inflammatory responses in patients [258]. Furthermore, MSCs have been shown to mitigate damage to the endothelial and epithelial barriers, thereby protecting lung function, as evidenced in clinical trials [259, 260]. Meanwhile, Extracellular vesicles (EVs) secreted by MSCs have emerged as a promising treatment modality for ALI/ARDS, attracting considerable attention. Advances in EV characterization methods have facilitated their study [261]. EVs use a cell-free therapeutic approach Moreover, MSC-derived EVs can transfer cellular contents to recipient cells, thereby reducing inflammation and oxidative stress and ultimately alleviating lung injury [262]. Additionally, the increase in EVs in human endothelial cells may promote angiogenesis and regulate immune responses [258]. Despite the promise of this cell-free approach, challenges persist in the field, necessitating the development of treatments that meet rigorous criteria.

## Conclusion

In this review, we comprehensively discussed recent research progress regarding the role of ECs in sepsis-induced ALI/ARDS, as well as their interactions with other cell types. Recent literature emphasizes the pivotal roles played by ECs in the pathogenesis of ALI/ARDS triggered by sepsis, posing a significant challenge to the clinical management of septic patients. EC dysfunction in LPS-induced ALI/ARDS contributes to glycocalyx degradation, inflammation, oxidative stress, and other pathological processes through various pathways, thereby exacerbating disease progression and impacting prognosis. Moving forward, it is essential to adopt mechanistically focused trial designs that prioritize organ and cellular function characteristics to evaluate the potential benefits of protective strategies targeting ECs in the clinical management of sepsis. Additionally, ongoing development of diagnostic methods capable of assessing EC function in clinical settings is urgently needed. Such methods would complement therapeutic interventions aimed at strengthening and restoring endothelial function, particularly in sepsis cases, facilitating the identification of more effective agents capable of targeting the endothelium.

## Data Availability

No datasets were generated or analysed during the current study.

## Abbreviations

ADAMs: A disintegrin and metalloproteinases
AIF: Apoptosis-inducing factor
AJs: Adherens junctions
ALI: Acute lung injury
Ang2: Angiopoietin-2
APC: Activated protein C
ARDS: Acute respiratory distress syndrome
BALF: Bronchoalveolar lavage fluid
CLP: Cecal ligation and puncture
CTGF: Connective tissue growth factor
CYP: Cytochrome P450
DAMPs: Damage-Associated Molecular Patterns
DIC: Disseminated intravascular coagulation
ECs: Endothelial cells
EGFR: Epidermal growth factor receptor
eNOS: Endothelial nitric oxide synthase
EPCR: Endothelial protein C receptor
EPCs: Endothelial progenitor cells
ET-1: Endothelin-1
EVs: Extracellular vesicles
FGF: Fibroblast growth factor
FAD: Flavin adenine dinucleotide
Fe-S: Iron-sulfur center
GAG: Glycosaminoglycan
GM-CSF: Granulocyte-macrophage colony-stimulating factor
HA: Hyaluronan
HMGB1: High mobility group box 1
HS: Heparan sulfate
ICAM-1: Intercellular cell adhesion molecule-1
ICU: Intensive care unit
IkB: NF-κB inhibitor
IKK: IkB kinase
iNOS: Inducible nitric oxide synthase
LPS: Lipopolysaccharide
MSCs: Mesenchymal stem cells
MAPK: Mitogen-activated protein kinase
MIF: Migration inhibitory factor
miR-126-3p: MicroRNAs-126-3p
MLC: Myosin light chain
MLCK: Myosin light chain kinase
MLCP: Myosin light chain phosphatase
MMPs: Matrix Metalloproteinases
NETs: Neutrophil extracellular traps
NLRP3: NOD-like receptor thermal protein domain associated protein 3
NO: Nitric oxide
NOX: NADPH oxidase
PAMPs: Pathogen-Associated Molecular Patterns
PAR-1: Protease-activated receptors − 1
PFKFB3: 6-phophofructo-2-kinase/fructose-2, 6-biphosphatase
PGI: Prostacyclin
PGs: Proteoglycans
PIK3R4: Phosphoinositide 3-kinase regulatory subunit 4
PPARγ: Peroxisome proliferator-activated receptorγ
rAT: Recombinant antithrombin
REDOX: Reduction/oxidation
Rho: Ras homology
RIP 3: Receptor-interacting protein 3
ROCK: Rho-associated coiled-coil–forming protein kinases
ROS: Reactive oxygen species
S1P: Sphingosine 1-phosphate
SIRS: Systemic inflammatory response syndrome
SIRT1: Silent information regulator sirtuin 1
SOCS: Suppressor of cytokine signaling
SPRED1: Sprouty-related EVH1 Domain 1
TF: Tissue factor
TFPI: Tissue factor pathway inhibitor
Tie: Tie receptor
TJs: Tight junctions
TLRs: Toll-like receptors
TM: Thrombomodulin
TNFR1: Tumor necrosis factor receptor-1
TNFR1: Tumor necrosis factor receptor 1
t-PA: Tissue-type plasminogen activator
TRAF6: Tumor necrosis factor receptor-associated factor 6
UA: Uric acid
u-PA: Urokinase-type plasminogen activator
VCAM-1: Vascular cell adhesion molecule-1
VE-cadherin: Vascular endothelial cadherin
VEGF: Vascular endothelial growth factor
vWf: Von Willebrand factor
XO: Xanthine oxidase
YAP: Yes-associated protein
